# Aging and haptic shape discrimination: the effects of variations in size

**DOI:** 10.1038/s41598-020-71894-y

**Published:** 2020-09-07

**Authors:** J. Farley Norman, Jessica M. Dukes, Tori N. Palmore

**Affiliations:** 1 Department of Psychological Sciences, Ogden College of Science and Engineering, Western Kentucky University, 1906 College Heights Blvd. #22030, Bowling Green, KY 42101-2030 USA; 2 Center for Applied Science in Health and Aging, Western Kentucky University, Bowling Green, KY 42101-2030 USA

**Keywords:** Sensory processing, Somatosensory system, Human behaviour

## Abstract

Seventy-two older and younger adults haptically discriminated the solid shape of natural objects (bell peppers, *Capsicum annuum*). Plastic copies of the original-sized fruits were used as experimental stimuli, as well as copies that were reduced in size to 1/8th and 1/27th of the original object volumes. If haptic object shape is represented in a part-based manner, then haptic shape discrimination performance should be at least partly size invariant, since changes only in scale do not affect an object’s constituent parts. On any given trial, participants sequentially explored two bell pepper replicas and were required to judge whether they possessed the same shape or had different shapes. For some participants, the objects to be discriminated possessed the same size, while for others, the two objects had different sizes. It was found that variations in scale did significantly reduce the participants’ haptic sensitivities to shape. Nevertheless, the discrimination performance obtained for large variations in size was no lower than that obtained for smaller variations in size. The results also demonstrated that increases in age modestly affect haptic shape discrimination performance: the d′ values of the older participants were 15.5% lower than those of the younger participants.

Almost 60 years ago, James Gibson^1,2^ developed a set of solid objects (sculptures referred to as “feelies”; also see Norman et al.^3^) to be used in investigations of haptic shape discrimination and matching. The quantitative data from the resulting haptic shape discrimination experiments (conducted by Gibson's graduate student James Caviness) appeared several years later^4^. Following a hiatus, interest and research in haptic shape discrimination continued over the subsequent decades^3,5–13^. In our own laboratory’s research^3,7,8,11–13^ on shape discrimination, we have often used sets of naturally-shaped objects (bell peppers, *Capsicum annuum*). One good reason to use such objects is that many (but not all) pairs of these bell peppers are confusable to human participants and those systematic confusions are informative; they help us to identify the object features that confusable objects possess in common. Consider Fig. 1, which illustrates a confusion matrix obtained from participants in Experiment 1 of Norman et al.^7^; responses along the diagonal indicate correct judgments, while off-diagonal responses indicate mistakes. Notice that when human participants are presented with bell pepper #1, they often respond (i.e., believe) that it is object 3; conversely, when participants are presented with bell pepper #3, they frequently believe it to be object 1. Since objects 1 and 3 are reciprocally confusable with each other, they presumably share featural similarities that are important (and salient) to human perceptual systems. It is important to note that while objects 1 and 3 are confusable with each other (and also with objects 7 and 8), neither of these objects are confused with object 5. Objects 1, 3, and 5 (top, middle, and bottom, respectively) are illustrated in Fig. 2. Over the years across a variety of studies^3,7,8,11–13^, some of our participants have spontaneously noted that objects 1 and 3 both possess a “forehead” and a “chin”, while object 5 looks like a clenched “fist” (containing multiple “fingers”). In other words, our participants often spontaneously indicate that these naturally-shaped objects possess parts that are distinctive to them; they confuse objects that have similar parts (and don’t confuse objects possessing different parts).

**Figure 1:**
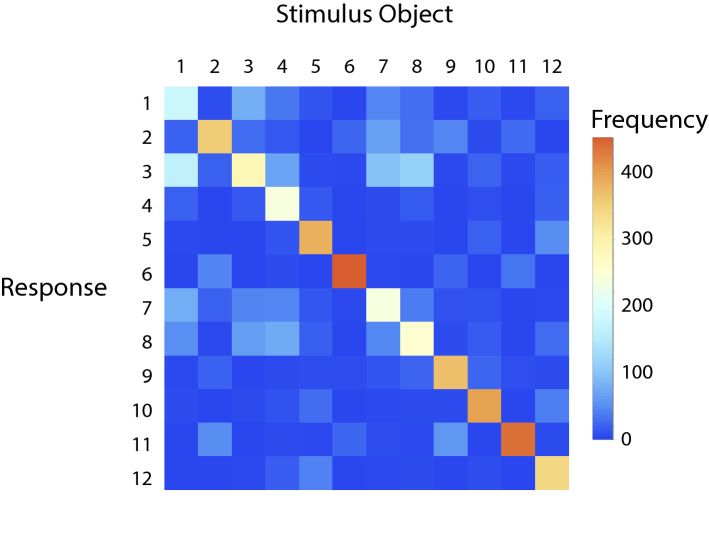
The confusion matrix of participant responses obtained from Experiment 1 of Norman et al.^7^. For each of the 12 stimulus objects (Bell peppers, *Capsicum annuum*) are plotted the frequencies of the 12 possible responses. Correct responses (e.g., stimulus 1 was identified as object 1; stimulus 12 was identified as object 12) are located along the diagonal. Cells colored dark blue indicate a frequency of zero for those stimulus–response pairs, while light blue, light cyan, yellow, orange, and red indicate higher and higher response frequencies. The off-diagonal light blue and cyan colored cells indicate participant confusions (note, for example, that when participants haptically explored stimulus object 1, they often believed it to be either object 3, object 7, or object 8; stimulus object 2 was often confused with objects 6, 9, and 11, etc.).

**Figure 2:**
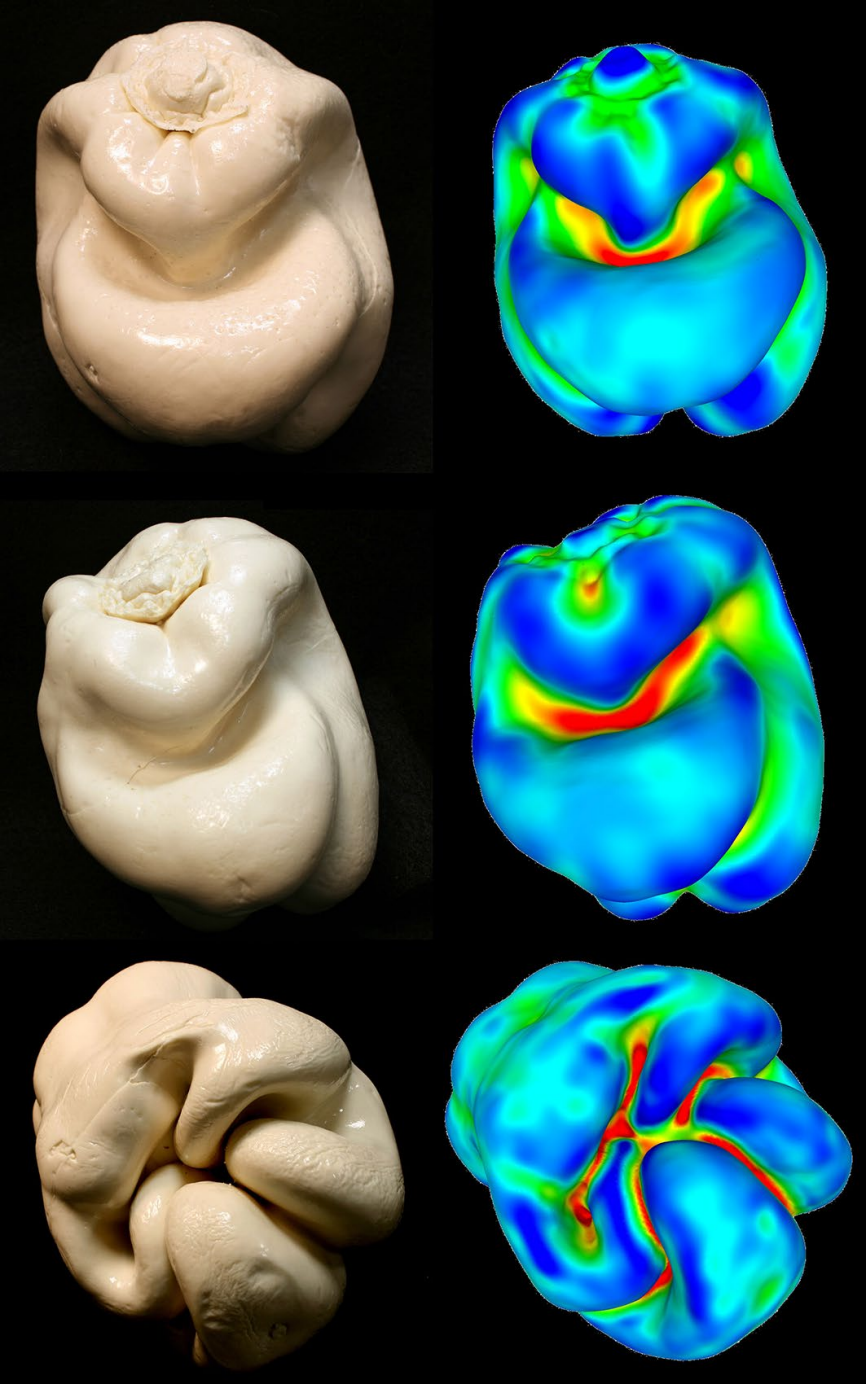
Photographs of stimulus objects 1, 3, and 5 are presented in the left panel (top, middle, and bottom, respectively). Plots of mean surface curvature for those same objects are presented in the right panel. Areas of lower mean curvature are colored blue and green, while higher local curvature magnitudes are colored yellow, orange, and red. The red surface regions indicate Hoffman and Richards’^17^ areas of maximal negative curvature (i.e., troughs), which separate the continuous object surfaces into parts. These photographs were taken by the first author, J. Farley Norman.

In his investigations with the feelies, Gibson^14^ found that in making their haptic judgments, participants gravitated towards concavities and troughs in the solid object surfaces. In particular, he said (p. 125) “when an observer is given one of these shapes to feel with his hands behind a curtain, he typically does the following things. (1) He curves his fingers around its face, using all fingers and fitting them into the cavities. (2) He moves his fingers in a way that can only be called exploratory, since the movements do not seem to become stereotyped, or to occur in any fixed sequence”. Our own experiences^3,7,8,11–13^ are consistent with Gibson’s observation. When handed one of our bell pepper stimuli (without vision), our participants also actively manipulate the stimulus objects; when they encounter a trough, groove, or other depression, they immediately stick their fingers into the concavity to explore its length, breadth, and shape. These observations are important, because they suggest that there is a critical similarity between vision and haptics in terms of how solid object shape is perceived and represented^15,16^. In 1984, Hoffman and Richards^17^ developed a theory of visual object recognition in which object shapes are represented in terms of parts (also see Singh and Hoffman^18^). Object surfaces are decomposed into parts wherever troughs occur; in particular, according to Hoffman and Richards^17^ the division into parts occurs (p. 74) “where, locally, the surface has greatest negative curvature”. Where do such surface locations exist for our bell pepper stimuli? Consider the right column of Fig. 2. The red surface areas indicate Hoffman and Richards’^17^ areas of maximal negative curvature (i.e., troughs), which separate the object surfaces into parts (we calculated the differential geometry and local surface curvatures for all regions on our object surfaces; areas of lesser mean curvature are colored blue and green, while higher local curvature magnitudes are colored yellow, orange, and red). One can see that, indeed, the troughs (red areas) do separate the “forehead” and “chin” parts of objects 1 and 3 and the “fingers” of the object (object 5) that our participants often call a clenched “fist”. The fact that our participants, in haptic experiments, often spontaneously refer to the parts of our objects in a manner that is consistent with Hoffman and Richards theory of visual perception and recognition is striking and suggests that perhaps haptic object perception (discrimination and recognition) is also based (at least partly) upon representations of parts. A significant body of evidence has accumulated to show that the theory of Hoffman and Richards has validity for vision^19,20^. Since visual and haptic information about objects is combined within the central nervous system^21,22^, if visual mechanisms are sensitive to discrete object parts, it would not be surprising if haptic mechanisms are also sensitive to parts.

If the haptic perception of shape, like that of vision, is based upon a part-based representation of objects (where the parts are separated by local minima, i.e., troughs), then perceived shape should remain essentially invariant with (reasonable) changes in object size^23^, since a change only in scale will not affect the parts (for example, see Fig. 3). One purpose of the current study was to examine this issue and determine whether and to what extent the haptic discriminability of solid object shape is actually affected by changes in scale. Previous research by Craddock and Lawson^10^ that utilized manmade objects and animal figurines (e.g., chairs, sinks, and bathtubs; horses and giraffes) suggests that shape discrimination will be substantially affected. However, other results (e.g., results of a visual study by Norman et al.^24^) have demonstrated a large degree of scale invariance. In a recent review provided by Lacey and Sathian^15^, while discussing the findings of Craddock and Lawson^10^, these authors (p. 6) pointed out “that haptic … and cross-modal … recognition are apparently size-dependent and this merits further investigation. Further research should address whether… object constancy can be achieved across size changes in unfamiliar objects.” We agree with Lacey and Sathian that further research is needed concerning how the haptic shape perception of unfamiliar objects is affected by variations in scale. An additional second purpose of the current study is to determine whether the effects of size manipulation documented by Craddock and Lawson generalize and extend to unfamiliar object stimuli (such as our individually-shaped bell peppers).

**Figure 3:**
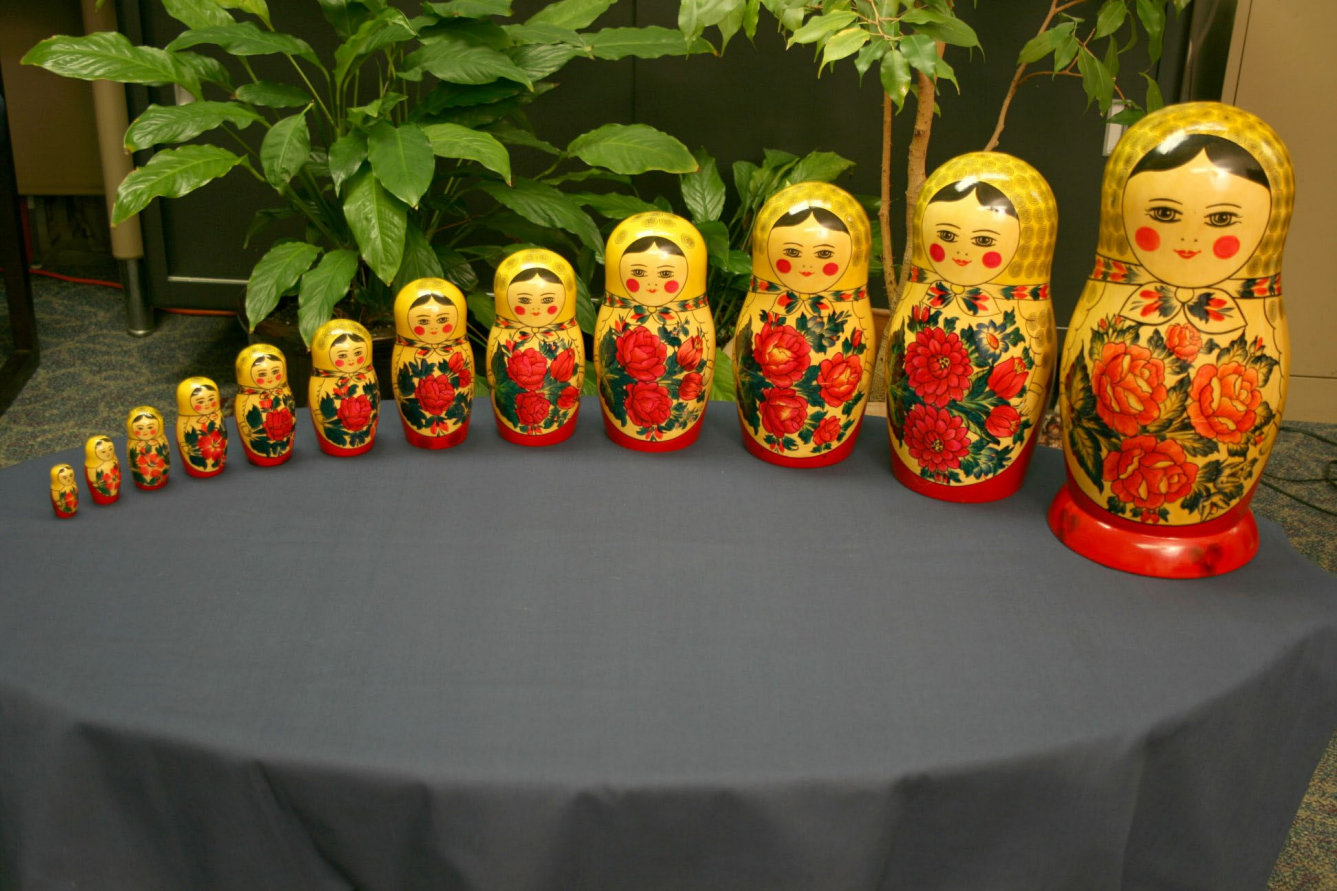
A photograph of a set of 12 wooden dolls. One can readily see that while the dolls vary widely in size, they all nevertheless possess the same shape. According to the minima rule of Hoffman and Richards^17^, all of these objects, regardless of their scale, possess two distinct parts (i.e., the “head” and the “body” are separated by a saddle-shaped trough; the single largest doll in this figure has an extra part, a supporting base). This photograph was taken by the first author, J. Farley Norman.

A third purpose of the current study was to evaluate potential effects of aging. Even if the haptic shape judgments (for unfamiliar objects) of younger adults are adversely affected by changes in object size, it is possible that the haptic judgments of older adults might be even more negatively affected because of age-related reductions in manual dexterity. In the current study, the full-sized natural objects (plastic replicas of bell peppers, *C. annuum*, see Fig. 4) were scaled down to one-eighth and one-twenty-seventh of their original size (see Fig. 5). Because older adults have poorer manual dexterity^25,26^ than younger adults, changes in size (especially reductions, like those employed in the current study) may have more detrimental effects upon their haptic performance.

**Figure 4:**
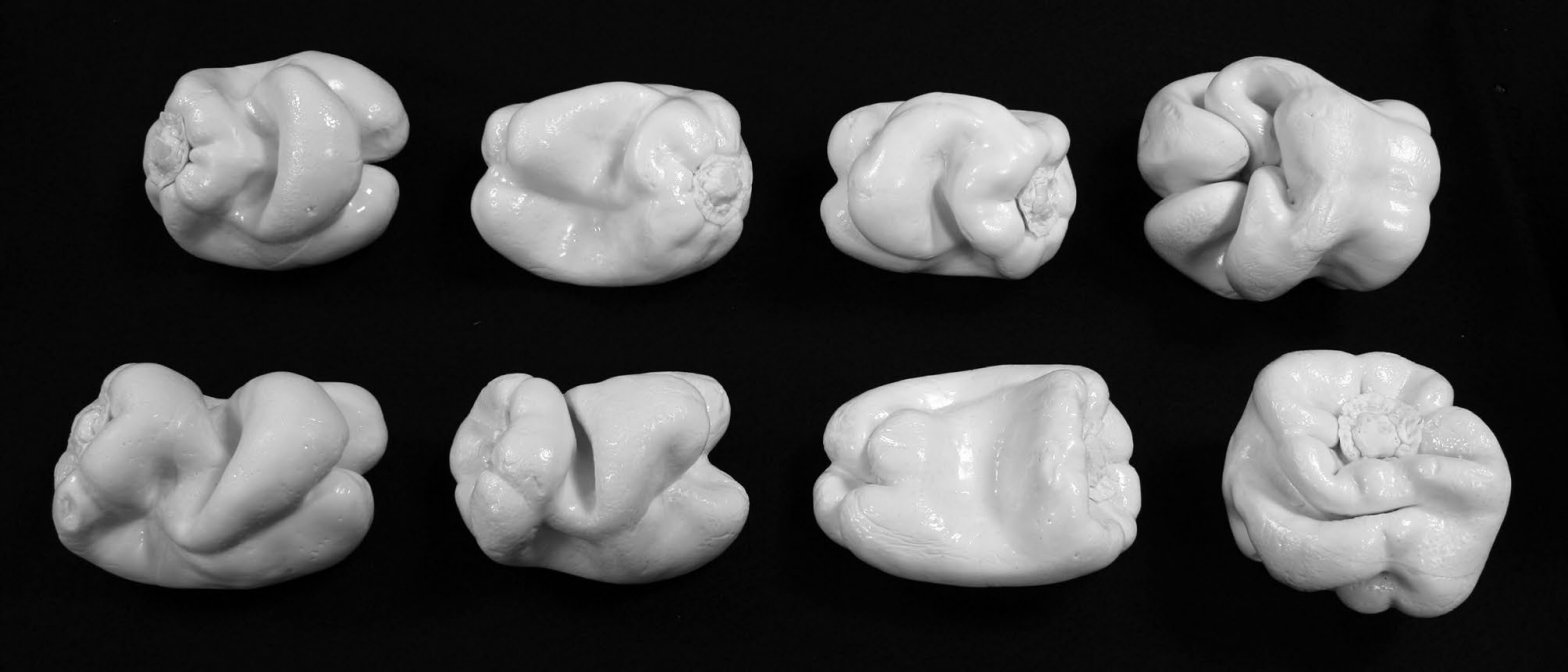
A photograph of the eight bell pepper (*Capsicum annuum*) replicas used as experimental stimuli. From upper-left to bottom-right, the objects are 1, 2, 3, 5, 7, 8, 11, and 12. The six different object pairs used in the current experiment (objects 1 and 3, objects 1 and 7, objects 2 and 11, objects 3 and 7, objects 3 and 8, and objects 5 and 12) represent the most confusable object pairs, as found by Norman, Norman, et al.^7^. This photograph was taken by the first author, J. Farley Norman.

**Figure 5:**
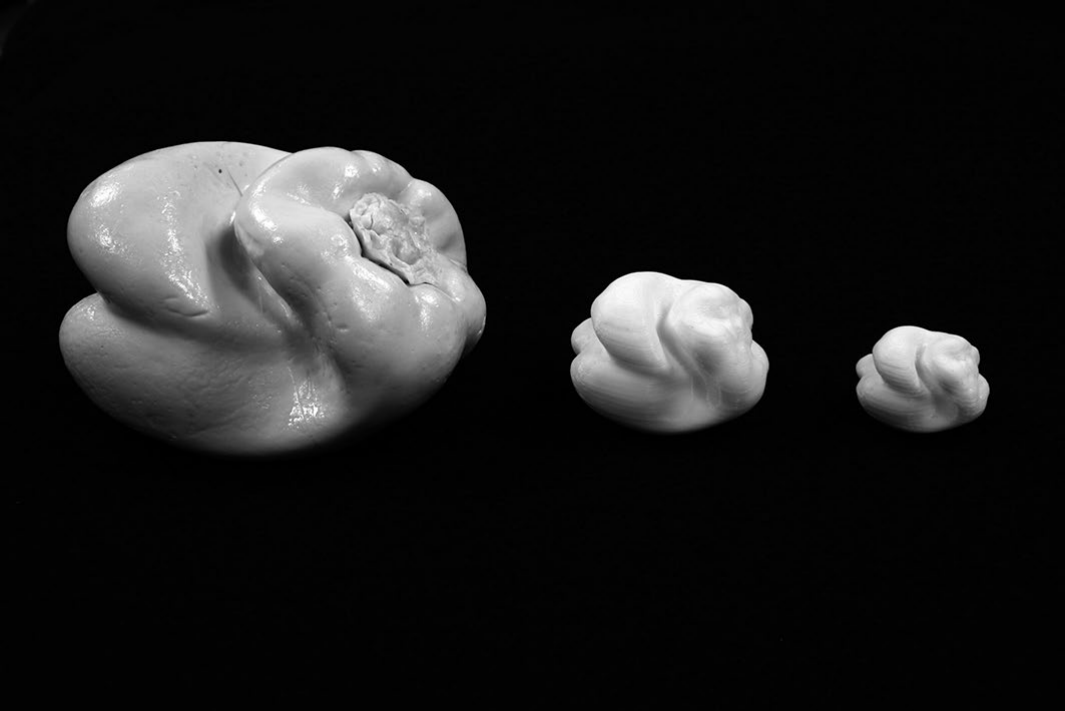
A photograph of bell pepper 3 reproduced at three sizes; from left to right are indicated the original (large) size, the medium size (1/8th of the original volume), and the small size (1/27th of the original volume). This photograph was taken by the first author, J. Farley Norman.

## Methods

### Apparatus

The medium (one-eighth volume) and small (one-twenty-seventh volume) sets of bell pepper replicas were printed in PLA plastic (polylactic acid) using a Bits From Bytes 3D Touch printer following Laser Scanning (NextEngine Laser Scanner) of the full-sized objects (see “Experimental stimuli” section). An Apple MacBook computer was used to randomly order the presentation of the experimental stimuli and record the participants’ responses.

### Experimental stimuli

The full-sized stimulus objects (exact plastic copies of 8 natural bell peppers, *C. annuum*) were a subset (objects 1, 2, 3, 5, 7, 8, 11, and 12) of those used in previous research^3,7,8^.

### Procedure

The basic procedures for the shape discrimination task were similar to those used by Experiment 2 of Norman et al.^7^, Experiment 1 of Norman et al.^8^ and Norman et al.^3^. On each trial, participants reached behind an occluding curtain to haptically explore two stimulus objects sequentially (presented for 3 s each, separated by a 3-s interstimulus interval, ISI). The objects were presented in a random orientation each time, and the participants used both hands to actively explore each object's shape. After both objects had been presented, the participants' task was to indicate whether the objects possessed the same shape or had different shapes (regardless of size). Each participant made a total of 96 shape discrimination judgments (48 *same trials*, and 48 *different trials*), where the order of the same and different pairs of objects was determined randomly. As in some of our previous investigations^11,12^, only the most difficult (i.e., most confusable, see Fig. 1) object pairs (objects 1 and 3, objects 1 and 7, objects 2 and 11, objects 3 and 7, objects 3 and 8, and objects 5 and 12) were presented to the participants during *different trials* (each of these 6 different object pairs was presented 8 times throughout the entire block of 96 trials; during each of the 48 *same trials*, one of the 8 stimulus objects was randomly selected and presented twice).

There were three between-subjects’ conditions. For some participants, the two objects to be discriminated on each trial possessed the same size (both were of the medium size). For two other groups of participants, they had to discriminate object shape irrespective of differences in size: one group judged objects that differed in size by *one step* (either medium and large or small and medium) while another group judged objects that differed in size by *two steps* (small and large objects). For the groups that judged pairs of objects that differed in size, the larger of the two objects (e.g., medium size for people who judged small and medium objects) was sometimes presented first (with 50% probability) and sometimes presented second (also with 50% probability).

In addition to object shape discrimination, we evaluated the participants’ manual dexterities using a modified version of the Moberg pick-up test^8,25–29^. For this task, the younger and older participants picked up 12 small metal objects (e.g., nail, paperclip, coins, flat-head screw, a key, a wing nut, etc.) and placed them one at a time into a container as fast as possible; the total time required to place the objects in the container was recorded. The participants performed this test both with and without vision. The participants’ dexterity was assessed twice (twice with vision and twice without); their best performance (i.e., fastest times) was included in the analysis.

### Participants

Seventy-two younger and older adults participated in the experiment (24 for each of the three between-subjects’ size conditions described earlier). Thirty-six of the participants were older (*M* = 73.5 years of age, *SD* = 4.9, range = 65 to 82 years) and 36 were younger (*M* = 21.5 years of age, *SD* = 2.5, range = 18 to 32 years). All participants were naive regarding the purpose of the experiment. The study was approved by the Institutional Review Board of Western Kentucky University, and each participant signed an informed consent document prior to testing. Our research was carried out in accordance with the Code of Ethics of the World Medical Association (Declaration of Helsinki).

## Results

**Figure 6:**
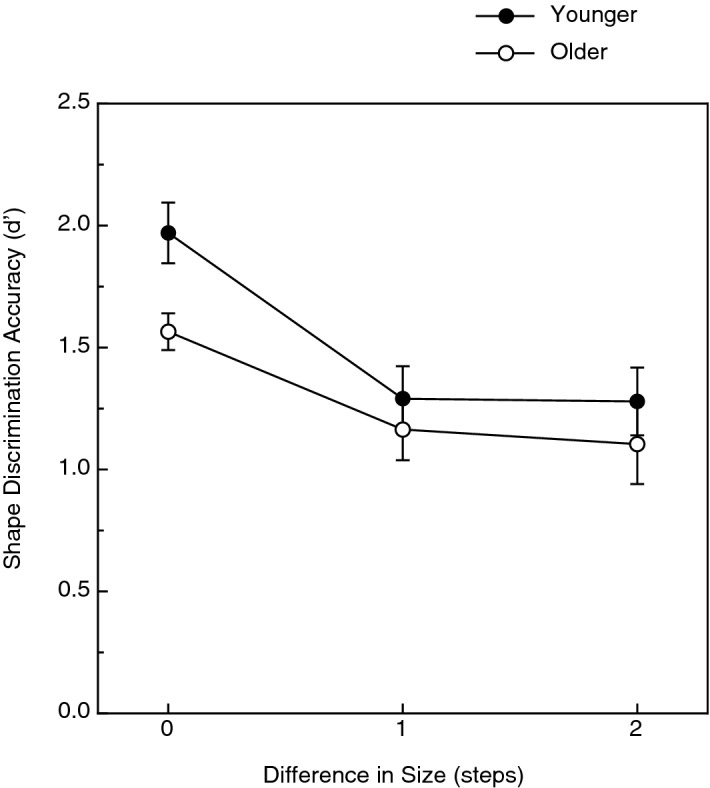
Experimental results. The younger and older participants’ haptic shape discrimination accuracies (d′ values) are plotted for all three of the size conditions. The error bars indicate ± 1 SE.

The results (d′ values, see Macmillan and Creelman^30^) for the younger and older participants are shown in Fig. 6. As is clearly evident from the figure, changes in size did indeed reduce shape discrimination performance when compared to the performance obtained for same-size objects (F(2, 66) = 12.4, *p* < 0.0001; η^2^_p_ = 0.27) as indicated by a 2-way between subjects analysis of variance (ANOVA). A Tukey HSD post-hoc test indicated that performance in the same-size condition was significantly different from that obtained in the one-step and two-step size change conditions. The magnitude of size change, however, did not matter: the performances obtained in the one-step and two-step size change conditions did not differ from each other (the performance obtained for small-large object pairs was no worse than that obtained for small-medium or medium-large object pairs). As is also evident from an examination of Fig. 6, there was a significant main effect of age (F(1, 66) = 4.9, *p* = 0.03; η^2^_p_ = 0.07); the younger participants’ shape discrimination performance was 18.4% higher than that exhibited by the older participants. The effect of the size change was similar for both age groups, however, as indicated by the lack of a significant interaction between age and size condition (F(2, 66) < 1.0, *p* = 0.52).

**Figure 7:**
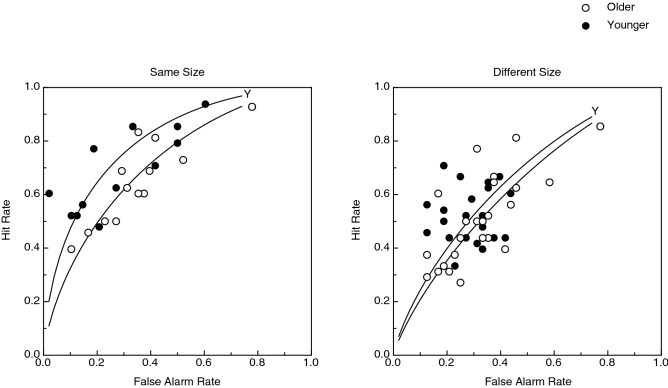
Experimental results. The participants’ hit rates and false alarm rates are presented for the same-size object condition (left panel) and the different-size object conditions (right panel). The hit- and false-alarm rates are plotted separately for the younger (filled circles) and older (open circles) participants. The participants’ ROC (receiver operating characteristic) curves are also plotted for each age group.

The participants’ hit rates (proportion of differently-shaped object pairs judged as different) and false alarm rates (proportion of same-shaped object pairs judged as different) are presented in Fig. 7 (the two different-size conditions, one-step and two-steps, have been combined, because there was no difference between them, see Fig. 6); the participants’ receiver operating characteristic (ROC) curves are plotted for each age group. The participants’ hit- and false alarm rates were subjected to two-way between-subjects’ ANOVAs. As can be seen from an inspection of Fig. 7, there was a significant effect of size condition upon the participants' hit rates (F(2, 66) = 9.9, *p* < 0.0002; η^2^_p_ = 0.23), such that the hit rates were significantly higher in the same size condition. Therefore, any variation in size made it more difficult for participants to detect that two objects differed in shape (when in fact they did differ in shape). Interestingly, there were no significant effects of age upon either hit or false alarm rates (e.g., no main effects of age; hit rate: F(1, 66) = 1.09, *p* = 0.30; false alarm rate: F(1, 66) = 0.80, *p* = 0.38).

**Figure 8:**
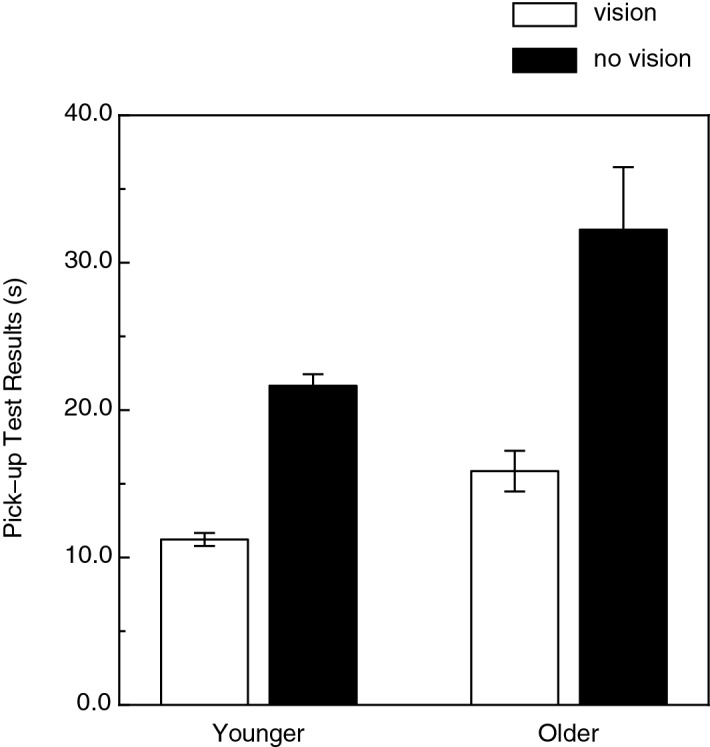
Experimental results (Manual dexterity). The results of the Moberg Pick-up Test are plotted for the younger and older participants. The cumulative pick-up times for the with- and without-vision conditions are indicated by the white and black bars, respectively. The error bars indicate ± 1 SE.

The results of the pick-up test of manual dexterity are shown in Fig. 8. As can clearly be seen in the figure, there were significant effects of both age and the presence/absence of vision (age: F(1, 70) = 7.5, *p* < 0.01, η^2^_p_ = 0.10; vision/no-vision: F(1, 70) = 68.8, *p* < 0.000001, η^2^_p_ = 0.50). The older participants were less dexterous than the younger participants (i.e., their pick-up times were 46.3% higher). Given that the outcome of the haptic shape discrimination and dexterity tests were similar (e.g., adverse effect of age for both tasks), it is interesting that there was no significant relationship between these tasks (e.g., the Pearson r correlation coefficients between d′ and the no-vision [i.e., haptic only condition] pick-up times were − 0.224 and − 0.086 for the same and different object size conditions, respectively). Even if these correlations had been statistically significant (*p* values were 0.29 and 0.56 for the same and different object size conditions, respectively), variations in our participants’ manual dexterity would have accounted for only 5.0 (0.224^2^) and 0.74 (0.086^2^) percent of the variance in their haptic shape sensitivities (i.e., d′ values).

## Discussion

In previous research involving visual solid shape discrimination, there were only very small effects of size change (i.e., scale) upon performance (e.g., see Norman et al.^24^). In contrast, Craddock and Lawson^10^ found substantial effects of size change upon the haptic discrimination of manmade objects and animal figurines. Our current results are similar to those of Craddock and Lawson and demonstrate that their finding extends to the haptic discrimination of a quite different and unfamiliar set of solid objects. It is interesting, however, that while some size-change hurts shape discrimination performance (see Fig. 6), having additional amounts of size-change does not result in further deteriorations in performance (e.g., in our study, the performance for comparisons of small and large objects was no worse than the performances obtained for small and medium or medium and large objects).

In an absolute sense, our older participants did well; they do possess effective haptic shape discrimination abilities: their d’ values were 84.5% of those of the younger participants (overall d′ values were 1.278 and 1.513 for the older and younger participants, respectively). Nevertheless, our study does reveal that there is a significant adverse effect of age upon haptic shape sensitivity (i.e., d′ values). In contrast, we found no systematic effects of age with regards to either hit- or false-alarm rates. This is an interesting outcome (that there was a significant effect of age upon d′, but not upon hit- or false-alarm rates). For any given older adult, therefore, they were less sensitive to differences in shape than a typical younger adult, but that reduced performance was due to either a lower hit rate or a higher false-alarm rate (which differed across older individuals).

The pattern that was obtained in the current experiment (that the older adults’ haptic shape discrimination performance was qualitatively similar to that exhibited by the younger adults, but they nevertheless possessed lower sensitivity) has been observed before for other haptic tasks. In 2016, Cheeseman et al.^26^ determined curvature discrimination thresholds for older and younger adults. In this study, there was always relative movement between the participants’ index finger and a curved (convex or concave curvatures) cylindrical block. In one condition (active), the participants actively felt the curved surface of the stimulus blocks; in the other condition (passive), the curved block translated underneath the participants’ fingertip. Thus, the tactile stimulation at the fingertip was similar (and dynamic) for both active and passive conditions. Cheeseman et al. found that the resulting curvature discrimination thresholds were superior for the passive condition. What is important to note for the present purposes is that the older adults in the Cheeseman et al. study showed the same qualitative pattern of results (see their Fig. 3) as the younger adults, despite possessing a lower overall sensitivity to curvature. Multiple studies with quite different tactile/haptic stimuli have now demonstrated that older adults, while possessing less overall sensitivity to curvature and shape, nevertheless produce results that are qualitatively the same as those of younger adults.

## Conclusion

Despite exhibiting modest declines in solid shape discrimination performance, older adults nevertheless retain effective haptic abilities.

## Data Availability

The datasets generated during and/or analyzed during the current study are available from the corresponding author on reasonable request.
